# (Disulfur dinitrido)triphenyl­anti­mony(V)

**DOI:** 10.1107/S1600536810009487

**Published:** 2010-03-17

**Authors:** Alexandra M. Z. Slawin, Paul G. Waddell, J. Derek Woollins

**Affiliations:** a Department of Chemistry, University of St Andrews, St Andrews KY16 9ST, Scotland

## Abstract

The title compound, [Sb(C_6_H_5_)_3_(N_2_S_2_)], contains a molecular entity that is very similar to that of the known polymorph of Sb(S_2_N_2_)Ph_3_ [Kunkel *et al.* (1997 ▶). *Z. Naturforsch. Teil B*, **52**, 193–198], differing only in the orientation of the phenyl rings. The bond order in the SNSN unit is S—N=S=N, consisting of one long S—N bond, an inter­mediate length N=S bond and a short S=N bond.

## Related literature

For  the polymorph crystallizing in space group *P*2_1_/*n*, see: Kunkel *et al.* (1997 ▶). For Pt(S_2_N_2_)(P*R*_3_)_2_ complexes with a similar bond order in the SNSN unit, see: Bates *et al.* (1986 ▶); Read *et al.* (2007 ▶).
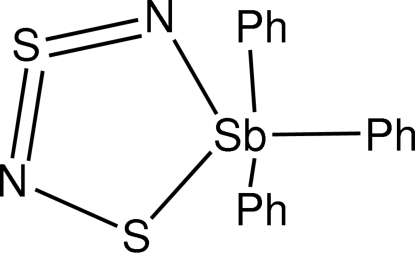


## Experimental

### 

#### Crystal data

[Sb(C_6_H_5_)_3_(N_2_S_2_)]
*M* *_r_* = 445.20
Monoclinic, 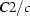

*a* = 16.997 (3) Å
*b* = 11.587 (2) Å
*c* = 18.166 (3) Å
β = 99.732 (7)°
*V* = 3526.3 (11) Å^3^
*Z* = 8
Mo *K*α radiation
μ = 1.80 mm^−1^
*T* = 93 K
0.08 × 0.05 × 0.05 mm

#### Data collection

Rigaku Mercury70 CCD diffractometer
Absorption correction: multi-scan (*ABSCOR*; Higashi, 1995 ▶) *T*_min_ = 0.741, *T*_max_ = 0.914
11155 measured reflections
3094 independent reflections
2826 reflections with *F* ^2^ > 2σ(*F* ^2^)
*R*_int_ = 0.053

#### Refinement

*R*[*F* ^2^ > 2σ(*F* ^2^)] = 0.051
*wR*(*F* ^2^) = 0.113
*S* = 1.37
3094 reflections
208 parameters
H-atom parameters constrained
Δρ_max_ = 3.38 e Å^−3^
Δρ_min_ = −1.67 e Å^−3^

### 

Data collection: *SCXMini* (Rigaku, 2006 ▶); cell refinement: *SCXMini*; data reduction: *SCXMini*; program(s) used to solve structure: *SHELXS97* (Sheldrick, 2008 ▶); program(s) used to refine structure: *SHELXL97* (Sheldrick, 2008 ▶); molecular graphics: *CrystalStructure* (Rigaku, 2009 ▶); software used to prepare material for publication: *CrystalStructure*.

## Supplementary Material

Crystal structure: contains datablocks global, I. DOI: 10.1107/S1600536810009487/jh2136sup1.cif

Structure factors: contains datablocks I. DOI: 10.1107/S1600536810009487/jh2136Isup2.hkl

Additional supplementary materials:  crystallographic information; 3D view; checkCIF report

## Figures and Tables

**Table 1:** Selected bond lengths (Å)

Sb1—C7	2.098 (6)
Sb1—C1	2.123 (6)
Sb1—C13	2.172 (5)
Sb1—N1	2.180 (5)
Sb1—S2	2.5030 (17)
S1—N1	1.510 (6)
S1—N2	1.589 (6)
S2—N2	1.656 (6)

**Fig. 1.**
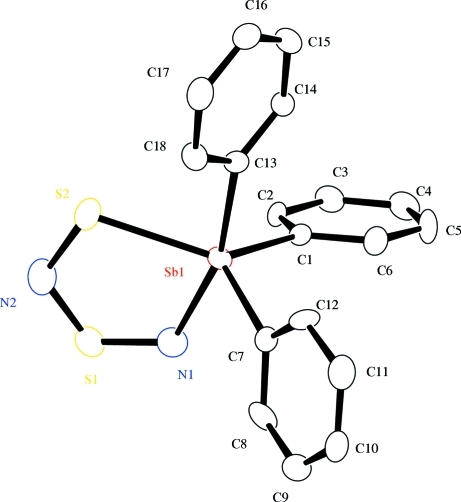
The structure of (1) with displacement ellipsoids drawn at the 50% probability level, hydrogen atoms omitted for clarity.

## supplementary crystallographic information

### Comment

Previously the title compound has been prepared from tetrasulfurteranitride. The synthesis described here uses a non explosive S—N starting material.

### Experimental

Liquid ammonia (30 ml) was condensed under nitrogen using an ammonia condenser filled with dry ice and acetone into a dry Schlenk tube in a dry ice/acetone bath. To this, 0.102 g (0.5 mmol) [S_4_N_3_]Cl was added. After stirring for 30 min s, 0.212 g (0.5 mmol) of triphenylstibenedichloride was added rapidly. The solution was then allowed to warm to room temperature and the ammonia gas blown off under a stream of nitrogen. The residue was placed under vacuum to remove any excess ammonia, before being dissolved in dichloromethane and filtered through celite. The product was precipitated by slow addition of hexane to give a yellow powder. Crystals were grown *via* slow diffusion of hexane into a solution of the product in dichloromethane.

### Refinement

All H atoms were included in calculated positions and refined as riding atoms with Uĩso~(H) = 1.5 U~eq~. The highest peak in the difference map is 0.83 Å from atom Sb1

### Crystal data

**Table:**

[Sb(C_6_H_5_)_3_(N_2_S_2_)]	*F*(000) = 1760.00
*M**_r_* = 445.20	*D*_x_ = 1.677 Mg m^−^^3^
Monoclinic, *C*2/*c*	Mo *K*α radiation, λ = 0.71070 Å
Hall symbol: -C 2yc	Cell parameters from 1987 reflections
*a* = 16.997 (3) Å	θ = 2.1–25.4°
*b* = 11.587 (2) Å	µ = 1.80 mm^−^^1^
*c* = 18.166 (3) Å	*T* = 93 K
β = 99.732 (7)°	Prism, yellow
*V* = 3526.3 (11) Å^3^	0.08 × 0.05 × 0.05 mm
*Z* = 8	

### Data collection

**Table:**

Rigaku Mercury70 CCD diffractometer	2826 reflections with *F*^2^ > 2σ(*F*^2^)
ω scans	*R*_int_ = 0.053
Absorption correction: multi-scan (*ABSCOR*; Higashi, 1995)	θ_max_ = 25.4°
*T*_min_ = 0.741, *T*_max_ = 0.914	*h* = −20→19
11155 measured reflections	*k* = −8→13
3094 independent reflections	*l* = −21→16

### Refinement

**Table:**

Refinement on *F*^2^	Secondary atom site location: difference Fourier map
*R*[*F*^2^ > 2σ(*F*^2^)] = 0.051	Hydrogen site location: inferred from neighbouring sites
*wR*(*F*^2^) = 0.113	H-atom parameters constrained
*S* = 1.37	*w* = 1/[σ^2^(*F*_o_^2^) + (0.0236*P*)^2^ + 20.1*P*] where *P* = (*F*_o_^2^ + 2*F*_c_^2^)/3
3094 reflections	(Δ/σ)_max_ = 0.002
208 parameters	Δρ_max_ = 3.38 e Å^−^^3^
0 restraints	Δρ_min_ = −1.67 e Å^−^^3^
Primary atom site location: structure-invariant direct methods	

### Special details

**Table:**

Geometry. ENTER SPECIAL DETAILS OF THE MOLECULAR GEOMETRY
Refinement. Refinement was performed using all reflections. The weighted *R*-factor (wR) and goodness of fit (*S*) are based on *F*^2^. *R*-factor (gt) are based on *F*. The threshold expression of *F*^2^ > 2.0 σ(*F*^2^) is used only for calculating *R*-factor (gt).

### Fractional atomic coordinates and isotropic or equivalent isotropic displacement parameters (Å^2^)

**Table:**

	*x*	*y*	*z*	*U*_iso_*/*U*_eq_	
Sb(1)	0.22035 (2)	0.02024 (4)	0.064344 (19)	0.0189	
S(1)	0.39390 (10)	0.02791 (16)	0.16524 (9)	0.0317	
S(2)	0.34568 (10)	0.05730 (17)	0.01298 (9)	0.0326	
N(1)	0.3060 (3)	0.0161 (5)	0.1682 (3)	0.0320	
N(2)	0.4187 (4)	0.0478 (6)	0.0857 (3)	0.0402	
C(1)	0.1547 (4)	0.1472 (5)	0.1125 (3)	0.0176	
C(2)	0.1924 (4)	0.2490 (5)	0.1401 (3)	0.0187	
C(3)	0.1493 (4)	0.3314 (6)	0.1715 (3)	0.0245	
C(4)	0.0709 (4)	0.3115 (6)	0.1773 (3)	0.0283	
C(5)	0.0330 (4)	0.2102 (6)	0.1503 (3)	0.0299	
C(6)	0.0750 (4)	0.1279 (6)	0.1179 (3)	0.0242	
C(7)	0.1724 (4)	−0.1403 (5)	0.0867 (3)	0.0210	
C(8)	0.1975 (4)	−0.1975 (6)	0.1561 (4)	0.0266	
C(9)	0.1631 (4)	−0.2976 (6)	0.1714 (4)	0.0327	
C(10)	0.1015 (4)	−0.3454 (6)	0.1208 (4)	0.0312	
C(11)	0.0752 (4)	−0.2915 (6)	0.0538 (4)	0.0319	
C(12)	0.1128 (4)	−0.1908 (5)	0.0364 (3)	0.0223	
C(13)	0.1639 (4)	0.0371 (5)	−0.0517 (3)	0.0192	
C(14)	0.1094 (4)	0.1244 (5)	−0.0747 (3)	0.0202	
C(15)	0.0780 (4)	0.1359 (6)	−0.1512 (3)	0.0266	
C(16)	0.1023 (4)	0.0618 (6)	−0.2029 (3)	0.0271	
C(17)	0.1570 (4)	−0.0245 (6)	−0.1798 (3)	0.0295	
C(18)	0.1880 (4)	−0.0367 (6)	−0.1046 (3)	0.0257	
H(2)	0.2471	0.2616	0.1374	0.022*	
H(3)	0.1740	0.4020	0.1890	0.029*	
H(4)	0.0423	0.3678	0.2002	0.034*	
H(5)	−0.0214	0.1976	0.1540	0.036*	
H(6)	0.0496	0.0583	0.0992	0.029*	
H(8)	0.2388	−0.1649	0.1919	0.032*	
H(9)	0.1813	−0.3358	0.2174	0.039*	
H(10)	0.0774	−0.4155	0.1325	0.037*	
H(11)	0.0319	−0.3226	0.0197	0.038*	
H(12)	0.0970	−0.1563	−0.0113	0.027*	
H(14)	0.0934	0.1758	−0.0393	0.024*	
H(15)	0.0400	0.1946	−0.1674	0.032*	
H(16)	0.0813	0.0705	−0.2545	0.033*	
H(17)	0.1734	−0.0753	−0.2154	0.035*	
H(18)	0.2259	−0.0957	−0.0889	0.031*	

### Atomic displacement parameters (Å^2^)

**Table:**

	*U*^11^	*U*^22^	*U*^33^	*U*^12^	*U*^13^	*U*^23^
Sb(1)	0.0204	0.0206	0.0152	0.0039	0.0013	−0.0005
S(1)	0.0231	0.0360	0.0332	−0.0013	−0.0035	0.0030
S(2)	0.0245	0.0462	0.0280	−0.0055	0.0072	0.0023
N(1)	0.0291	0.0398	0.0255	−0.0014	0.0001	0.0023
N(2)	0.0266	0.0576	0.0363	−0.0008	0.0053	0.0008
C(1)	0.0186	0.0233	0.0114	0.0019	0.0040	0.0012
C(2)	0.0136	0.0234	0.0201	−0.0021	0.0057	0.0017
C(3)	0.0273	0.0242	0.0207	0.0028	0.0004	−0.0023
C(4)	0.0345	0.0328	0.0179	0.0122	0.0050	−0.0038
C(5)	0.0143	0.0493	0.0275	0.0021	0.0077	−0.0031
C(6)	0.0216	0.0289	0.0230	−0.0016	0.0062	−0.0022
C(7)	0.0199	0.0203	0.0240	0.0014	0.0072	0.0010
C(8)	0.0213	0.0191	0.0362	0.0045	−0.0043	−0.0004
C(9)	0.0301	0.0348	0.0339	0.0066	0.0076	0.0102
C(10)	0.0240	0.0273	0.0458	0.0019	0.0160	0.0052
C(11)	0.0248	0.0270	0.0446	−0.0052	0.0075	−0.0039
C(12)	0.0307	0.0186	0.0167	−0.0061	0.0010	0.0043
C(13)	0.0203	0.0188	0.0180	−0.0018	0.0017	0.0019
C(14)	0.0174	0.0243	0.0184	−0.0010	0.0014	0.0010
C(15)	0.0213	0.0313	0.0255	0.0030	−0.0013	0.0053
C(16)	0.0246	0.0411	0.0141	−0.0056	−0.0009	0.0033
C(17)	0.0236	0.0429	0.0220	−0.0054	0.0037	−0.0067
C(18)	0.0235	0.0358	0.0178	0.0051	0.0029	−0.0016

### Geometric parameters (Å, °)

**Table:**

Sb1—C7	2.098 (6)	C8—C9	1.350 (9)
Sb1—C1	2.123 (6)	C8—H8	0.9500
Sb1—C13	2.172 (5)	C9—C10	1.387 (10)
Sb1—N1	2.180 (5)	C9—H9	0.9500
Sb1—S2	2.5030 (17)	C10—C11	1.375 (9)
S1—N1	1.510 (6)	C10—H10	0.9500
S1—N2	1.589 (6)	C11—C12	1.392 (9)
S2—N2	1.656 (6)	C11—H11	0.9500
C1—C6	1.392 (8)	C12—H12	0.9500
C1—C2	1.395 (8)	C13—C14	1.387 (8)
C2—C3	1.383 (8)	C13—C18	1.398 (8)
C2—H2	0.9500	C14—C15	1.407 (8)
C3—C4	1.374 (9)	C14—H14	0.9500
C3—H3	0.9500	C15—C16	1.387 (9)
C4—C5	1.388 (10)	C15—H15	0.9500
C4—H4	0.9500	C16—C17	1.381 (10)
C5—C6	1.382 (9)	C16—H16	0.9500
C5—H5	0.9500	C17—C18	1.385 (8)
C6—H6	0.9500	C17—H17	0.9500
C7—C12	1.375 (9)	C18—H18	0.9500
C7—C8	1.425 (8)		
			
C7—Sb1—C1	106.6 (2)	C8—C7—Sb1	121.1 (5)
C7—Sb1—C13	98.3 (2)	C9—C8—C7	120.5 (6)
C1—Sb1—C13	99.3 (2)	C9—C8—H8	119.8
C7—Sb1—N1	92.2 (2)	C7—C8—H8	119.8
C1—Sb1—N1	88.8 (2)	C8—C9—C10	120.8 (6)
C13—Sb1—N1	164.3 (2)	C8—C9—H9	119.6
C7—Sb1—S2	127.40 (17)	C10—C9—H9	119.6
C1—Sb1—S2	125.09 (17)	C11—C10—C9	120.1 (7)
C13—Sb1—S2	83.53 (16)	C11—C10—H10	120.0
N1—Sb1—S2	80.79 (15)	C9—C10—H10	120.0
N1—S1—N2	117.5 (3)	C10—C11—C12	119.3 (6)
N2—S2—Sb1	105.2 (2)	C10—C11—H11	120.3
S1—N1—Sb1	119.2 (3)	C12—C11—H11	120.3
S1—N2—S2	117.1 (4)	C7—C12—C11	121.4 (6)
C6—C1—C2	120.3 (5)	C7—C12—H12	119.3
C6—C1—Sb1	120.2 (4)	C11—C12—H12	119.3
C2—C1—Sb1	119.5 (4)	C14—C13—C18	119.8 (5)
C3—C2—C1	119.2 (6)	C14—C13—Sb1	121.4 (4)
C3—C2—H2	120.4	C18—C13—Sb1	118.7 (4)
C1—C2—H2	120.4	C13—C14—C15	119.3 (6)
C4—C3—C2	120.3 (6)	C13—C14—H14	120.4
C4—C3—H3	119.8	C15—C14—H14	120.4
C2—C3—H3	119.8	C16—C15—C14	120.2 (6)
C3—C4—C5	120.9 (6)	C16—C15—H15	119.9
C3—C4—H4	119.6	C14—C15—H15	119.9
C5—C4—H4	119.6	C17—C16—C15	120.3 (5)
C6—C5—C4	119.5 (6)	C17—C16—H16	119.8
C6—C5—H5	120.3	C15—C16—H16	119.8
C4—C5—H5	120.3	C16—C17—C18	119.8 (6)
C5—C6—C1	119.8 (6)	C16—C17—H17	120.1
C5—C6—H6	120.1	C18—C17—H17	120.1
C1—C6—H6	120.1	C17—C18—C13	120.6 (6)
C12—C7—C8	117.8 (6)	C17—C18—H18	119.7
C12—C7—Sb1	121.0 (4)	C13—C18—H18	119.7
			
C7—Sb1—S2—N2	−81.8 (3)	S2—Sb1—C7—C12	−97.6 (5)
C1—Sb1—S2—N2	85.8 (3)	C1—Sb1—C7—C8	−83.7 (5)
C13—Sb1—S2—N2	−177.4 (3)	C13—Sb1—C7—C8	174.0 (5)
N1—Sb1—S2—N2	3.8 (3)	N1—Sb1—C7—C8	5.7 (5)
N2—S1—N1—Sb1	2.4 (5)	S2—Sb1—C7—C8	85.8 (5)
C7—Sb1—N1—S1	124.0 (4)	C12—C7—C8—C9	−0.4 (9)
C1—Sb1—N1—S1	−129.4 (4)	Sb1—C7—C8—C9	176.4 (5)
C13—Sb1—N1—S1	−8.1 (11)	C7—C8—C9—C10	−1.5 (10)
S2—Sb1—N1—S1	−3.6 (3)	C8—C9—C10—C11	0.7 (10)
N1—S1—N2—S2	1.6 (6)	C9—C10—C11—C12	2.1 (10)
Sb1—S2—N2—S1	−3.9 (5)	C8—C7—C12—C11	3.2 (10)
C7—Sb1—C1—C6	−30.4 (5)	Sb1—C7—C12—C11	−173.6 (5)
C13—Sb1—C1—C6	71.2 (5)	C10—C11—C12—C7	−4.1 (10)
N1—Sb1—C1—C6	−122.3 (5)	C7—Sb1—C13—C14	115.8 (5)
S2—Sb1—C1—C6	159.8 (4)	C1—Sb1—C13—C14	7.3 (5)
C7—Sb1—C1—C2	148.3 (4)	N1—Sb1—C13—C14	−112.8 (8)
C13—Sb1—C1—C2	−110.0 (4)	S2—Sb1—C13—C14	−117.3 (5)
N1—Sb1—C1—C2	56.4 (4)	C7—Sb1—C13—C18	−69.1 (5)
S2—Sb1—C1—C2	−21.5 (5)	C1—Sb1—C13—C18	−177.6 (5)
C6—C1—C2—C3	−1.2 (8)	N1—Sb1—C13—C18	62.3 (10)
Sb1—C1—C2—C3	−180.0 (4)	S2—Sb1—C13—C18	57.9 (5)
C1—C2—C3—C4	1.9 (9)	C18—C13—C14—C15	1.2 (9)
C2—C3—C4—C5	−1.7 (9)	Sb1—C13—C14—C15	176.2 (4)
C3—C4—C5—C6	0.8 (9)	C13—C14—C15—C16	−1.0 (9)
C4—C5—C6—C1	−0.1 (9)	C14—C15—C16—C17	0.6 (10)
C2—C1—C6—C5	0.3 (9)	C15—C16—C17—C18	−0.3 (10)
Sb1—C1—C6—C5	179.1 (4)	C16—C17—C18—C13	0.5 (10)
C1—Sb1—C7—C12	93.0 (5)	C14—C13—C18—C17	−0.9 (10)
C13—Sb1—C7—C12	−9.4 (6)	Sb1—C13—C18—C17	−176.1 (5)
N1—Sb1—C7—C12	−177.6 (5)		
